# Antler cannibalism in reindeer

**DOI:** 10.1038/s41598-020-79050-2

**Published:** 2020-12-17

**Authors:** Atle Mysterud, Bjørnar Ytrehus, Michael A. Tranulis, Geir Rune Rauset, Christer M. Rolandsen, Olav Strand

**Affiliations:** 1 Centre for Ecological and Evolutionary Synthesis (CEES), Department of Biosciences, University of Oslo, Blindern, P.O. Box 1066, 0316 Oslo, Norway; 2 Norwegian Institute for Nature Research (NINA), Torgarden, P. O. Box 5685, 7485 Trondheim, Norway; 3 Department of Preclinical Sciences and Pathology, Norwegian University of Life Sciences, P.O. Box 369 Sentrum, 0102 Oslo, Norway

**Keywords:** Ecology, Zoology

## Abstract

Prion diseases constitute a class of invariably fatal and degenerative encephalopathies. Chronic Wasting Disease (CWD) is a contagious prion disease among cervids, which is spreading and causing marked population declines in USA and Canada. The first outbreak of CWD in Europe was discovered in a reindeer population in Norway in 2016. In the worst-case scenario with continental-wide spreading of CWD in Eurasia, an annual harvest of around 4 million cervids is at stake only in Europe, with huge economic and cultural significance. An in situ origin of CWD was suspected, and it appear urgent to identify the likely cause to prevent future emergences. Here, we document the novel phenomenon of extensive antler cannibalism prior to shedding among reindeer in the CWD-infected population. The extent of antler cannibalism increased over the last decades when CWD emerged, and included ingestion of vascularized antlers. Ingestion of tissues from conspecifics is a risk factor for the emergence of prion diseases, where the presence of extensive antler cannibalism opens the intriguing possibility of a ‘Kuru-analogue’ origin of CWD among the reindeer in Europe. Based on general insight on pathology of prion diseases and strain selection processes, we propose an hypothesis for how contagious CWD may emerge from sporadic CWD under the unique epidemiological conditions we document here. More research is required to document the presence of prions in reindeer antlers, and whether antler cannibalism actually led to a strain selection process and the emergence of a contagious form of CWD from a sporadic form of CWD.

## Introduction

Prion diseases comprise a group of incurable neurodegenerative maladies that affect several mammalian species, including humans^1^. In these diseases, the host-encoded cellular prion protein (PrP^C^) is misfolded into pathogenic variants (PrP^Sc^ scrapie conformers), of which the oligomeric aggregates create transmissible prion agents that induce a cascade of PrP^C^ misfolding. Prion diseases can be divided into sporadic, inherited, and contagious forms based on the epidemiology and pathogenesis^1^. In the infectious forms, such as in classical scrapie that infects sheep and goats, prions are present at high levels outside the central nervous system (CNS). This feature facilitates the release of prions to the surroundings with inter-individual (horizontal) transmissions under natural conditions. In the sporadic forms, the initial misfolding of PrP^C^ is suggested to occur spontaneously or as a consequence of a somatic mutation, with prions restricted to the CNS, which is indicative of limited contagiousness^2^. However, the boundaries between the forms are not strict. The ‘mad cow disease’, also known as bovine spongiform encephalopathy (BSE), most likely started when brain material from an animal with a sporadic prion disease was introduced to the bovine food chain, allowing an oral infection of conspecifics to occur^3^. Continued feeding of cattle with ruminant-derived protein, which contained incompletely inactivated prions of bovine origin, allowed the efficient spread of the pathogen^4^. The Kuru epidemic among members of the Fore tribe in Papua New Guinea has similarly been suggested to originate from the ingestion of brain material containing prions from a patient suffering from the sporadic case of Creutzfeldt Jakob disease^5^, which is a human prion disease of worldwide distribution. The infectious prion agent, found in large amounts in the central nervous tissue, was effectively transmitted to the villagers that participated in cannibalistic funeral rituals, causing new deaths and a dramatic escalation of the epidemic^6^. Chronic wasting disease (CWD) is a highly contagious prion disease that occurs among cervids, which is causing devastating economic and cultural impacts as the epizootic continues to grow in North America^7^. The emergence of CWD among reindeer (*Rangifer tarandus*) in Norway in 2016, came as a complete surprise to scientists and managers^8^, leading to a decision of population eradication and making headlines in *Nature*^9^ and *Science*^10^. The emergence of this disease was regarded as one of the latest and most important global conservation issues in a 2018 horizon scan^11^. Possible causes of the emergence of CWD included the introduction from the USA, or through ingestion of infectious tissue from the carcass of an individual with sporadic disease^8^. Strain typing identified a novel form of CWD prions from reindeer samples, which is different from the CWD prion strains found in North America^12^. This may indicate an in situ emergence in Norway, though strain selection of North American CWD strains for local prion protein genotypes (*PRNP*) cannot be disregarded.

Here, we documented the unique and bizarre phenomenon of extensive gnawing of intact antlers among the reindeer of the affected population in Norway. We provided comprehensive visual evidence using photographs and video footage of the phenomenon, while quantifying the frequency of antler cannibalism in the reindeer population where CWD emerged at the time of culling and in the decades prior to the emergence. We quantified the extent of antler cannibalism based on the data captured from the photographs of both the antlers and reindeer taken during the culling of this population and other reindeer populations (Table 1). We also discussed the likelihood of whether this has played a role in CWD emergence.

**Table 1:** The severity level of antler gnawing in populations of wild reindeer in Norway.

Population	Year	Data source	Level of antler gnawing (%)				Shed antler (n)	n
			None-low	Medium	High	Extreme		
**A. Southern metapopulation**								
Nordfjella (CWD)	2018	Photo of culled deer	3.4^a^	28.6	43.7	24.4		238
	2009	Photo from ground	27.6	13.8	22.4	36.2		58
	1984	Photo from ground	92.3	1.9	1.9	3.8		52
Hardangervidda	2020	Video from ground	3.4	1.4	2.0	93.2	57	350
	2004–2006	Photo from ground	7.3	9.3	15.2	68.2	5	156
**B. Northern metapopulation**								
Rondane	2009	Photo from GPS collars	44.2	15.4	18.3	22.1		104
Forollhogna	2019	Game camera	97.0	1.2	0.6	1.2		168
	2019	Video from ground	96.8	1.1	1.1	1.1	19	112

The levels were classified as none-low (no sign of gnawing or only antler tine ends were gnawed), medium (markedly gnawed, antler tines were clearly shortened), high (extensive gnawing, only main antler beams were left, with tines shorter than 2 cm or absent), and extreme (only a stump was left). All data from end of winter. Individuals with shed antlers were excluded from the calculation.

^a^All were in the low category with signs of antler gnawing.

## Results

**Figure 1:**
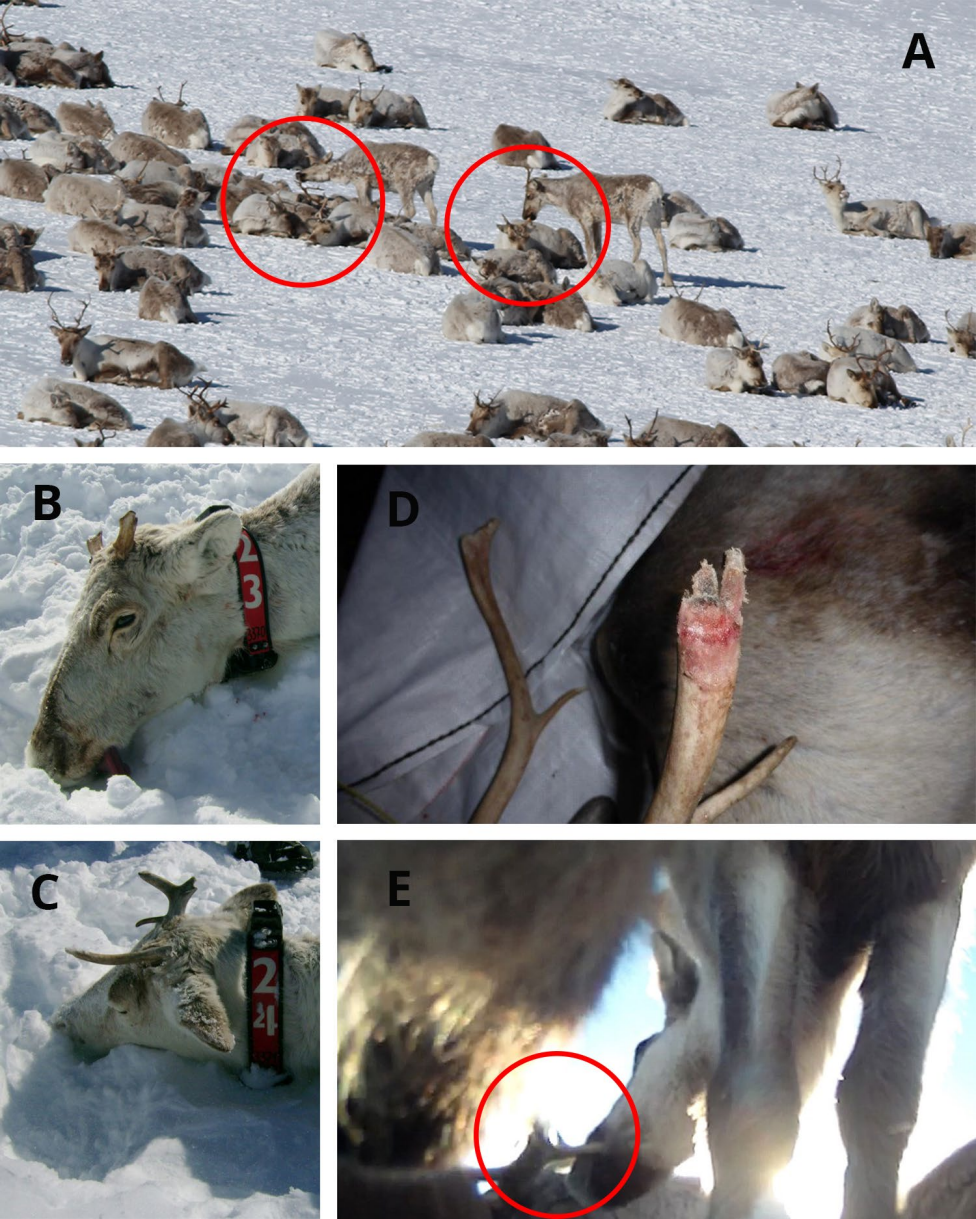
(**A**) Two upright female reindeer gnawing on another female reindeer’s antlers that was bedded down (Photo: Peter C.A. Köller). (**B,C**) Examples of antler stumps on female reindeer with a global positioning system (GPS)-collar that was used for scientific purposes (Photo: Roy Andersen). (**D**) Example of the gnawing that took place on the reindeer antler in Nordfjella (zone 1), Norway, when the antler was still vascularized (Photo: Lars Nesse). (**E**) Screenshot from supplementary video 1 showing antler gnawing filmed with camera on the GPS-collar.

**Figure 2:**
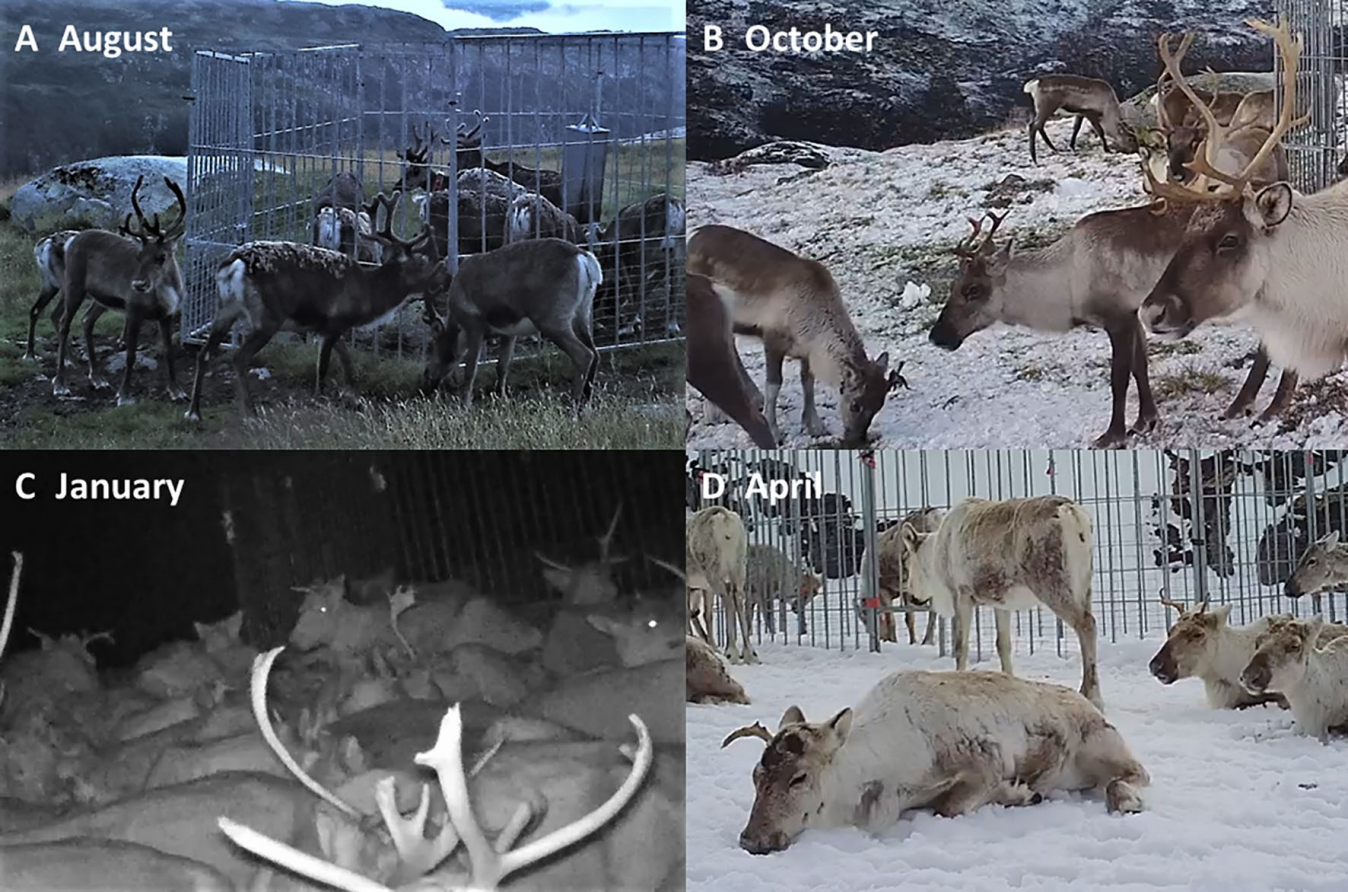
The phenology of antler gnawing in the reindeer population in Nordfjella (zone 2), Norway, based on camera trapping data at mineral licks. (**A**) Intact antlers in velvet in late summer (12. Aug. 2018). (**B**) Early sign of gnawing in fall (6. Oct. 2018). (**C**) Marked signs of gnawing in winter (5. Jan. 2019). (**D**) Extreme levels of antler gnawing evident in spring before shedding (20. Apr. 2019).

Antler cannibalism was found to be widespread and often left animals with their antlers gnawed down to the pedicle (Fig. 1, Table 1). We found that all the examined reindeer in the CWD-infected population of Nordfjella (zone 1) at the time of culling in 2018 showed signs of antler gnawing (n = 238), which extended from the visible signs of gnawing at the tine tips to the full gnawing of all the beams. In total, 43.7% had high levels of antler gnawing, while 24.4% showed extreme levels of gnawing, with only the antler stumps left and no tines on at least one side. The examined population mainly consisted of females (1 to 11 years old) and younger males (up to 4 years old), as older males would have shed their antlers at this time in February, 2018. Males had less gnawing than females, where the level of gnawing declined slightly with increasing age (Supplementary Figure 1, Supplementary Table 1). Based on the analyses of the photos from 1984 and 2009, the level of antler cannibalism has increased markedly in Nordfjella. In 1984, the vast majority (92.3%) of the reindeer in Nordfjella showed none or low level signs of gnawing. This proportion had declined markedly in 2009 and further in 2018 when only 3.4% had low levels (Table 1, Supplementary Table 2). The level of antler cannibalism was quite high in Hardangervidda being part of the same southern metapopulation, while in the northern metapopulation of reindeer, it was high in one population and absent in another (Table 1). We documented the actual gnawing behaviour using a global positioning system (GPS)-marked reindeer with a mounted camera on the collar (Supplementary Video 1, Fig. 1E), and camera traps at mineral licks in the adjacent population in Nordfjella zone 2 (Supplementary Video 2). The camera traps also documented the phenology of antler gnawing from intact antlers in August, through stages of increasing sign of gnawing during fall and early winter, ending in high to extreme levels in early spring before shedding (Fig. 2).

## Discussion

Osteophagia, which is the scientific term for bone and antler consumption, is a well-known phenomenon among ungulates in general^13,14^. However, it typically involves gnawing and the consumption of shed antlers or bone from carcasses. Such consumption is commonly attributed to mineral deficiencies^13,14^. Similarly, the act of geophagy^15,16^, ingestion of soil, predation, and carnivory^17^ are quite common behaviours among herbivores during periods of mineral deficiency. Our study documented, for the first time, the extensive cannibalism of intact antlers among reindeer. This phenomenon was discovered due to that the reindeer populations are now under much more scrutiny due to emergence of CWD.

### Patterns of antler gnawing

Antlers are unique to the male members of the *Cervidae* family, except in the case of reindeer and caribou, where females also grow antlers. Antler growth is energetically costly^18^. Male antlers are considered a secondary sexual characteristic^19^, where the signal of its portrayed dominance evolved from sexual selection. In the case of female antlers, their annual cycle and function both differ from that of males, where they play a role in the competition over grazing pits in the snow^20^. Females retain antlers during the winter, thereby gaining dominance over adult males that shed their antlers in early winter. Hence, antler cannibalism was expected to lower the dominance of the affected females. Younger males with antlers in the examined population showed less evidence of gnawing, while the level of gnawing was higher in younger females (Supplementary Figure 1). This may suggest a social component of antler cannibalism following the dominance hierarchy, but the role that initial antler size differences played in the rapid gnawing could not be disregarded.

### Antler gnawing and exposure to prions

In an anthropological study of human artefacts published in *Nature* in 1973, it was noted that: "Gaare (personal communication) records that southern Norwegian reindeer not only commonly chew shed antlers, but sometimes, also those on other reindeer”^13^. Antler cannibalism has therefore likely existed in this region for decades, but we presented evidence that high levels of antler gnawing increased from 1984 to now (Table 1, Supplementary Table 1). Hence, the behaviour was present and increased within the time frame of when CWD most likely had emerged. CWD prions were documented in elk (*Cervus canadensis*) antler velvet in North America^21^. In our study, we documented cases of antler cannibalism already occurring when antler bone still was vascularised (Fig. 1D). If ingestion of a reindeer’s antlers with sporadic prion disease was the event that initiated the outbreak of CWD in Norway, prions would have to be present in the antlers. Antlers show an impressive growth rate of up to 2 to 3 cm per day, where growing antler tissue is well innervated with sensory nerves originating from the trigeminal nerve^22^, i.e., cranial nerve V. Infective prions (PrP^Sc^) were found in the trigeminal ganglia of patients with Creutzfeldt-Jacobs disease^23^ and in other tissues innervated by the trigeminal nerve (cornea and gingiva). Antler bones remain viable and well perfused with blood vessels for several months after velvet shedding^24^. Bone marrow from Creutzfeldt-Jacobs disease patients also showed signs of high infectivity^25^. This suggests that gnawing on hard antlers from a reindeer with sporadic prion disease can constitute a risk factor to prion exposure long after velvet shedding (Fig. 1D). We have not yet documented prions in antlers from reindeer.

### Antler gnawing: any link to CWD?

In sporadic prion diseases, prions are largely confined to the CNS, while in contagious forms, such as classical scrapie and CWD, prions can be prominently present in peripheral lymphoid organs, including lymph nodes, spleen, and along the gastrointestinal tract^26^. Of the 19 reindeer that tested positive for CWD in Norway, all were positive in their lymphoid tissues^27^, therefore, contagious under natural conditions. Hence, in contrast to the Kuru epidemic among humans that was driven by ritual cannibalism, the transmission of CWD among reindeer in Norway likely involved common transmission mechanisms as experienced in North America. It is not known whether contagious prion diseases emerged and evolved from sporadic cases, where it would be required for prions from the CNS to enter the lymphatic tissue. Published literature open for this as a plausible scenario (overview in Table 2, Supplementary Note). The unusual features of the CWD variant seen in moose (*Alces alces*) in Norway^28^, Sweden and Finland, and a red deer (*Cervus elaphus*) in Norway^29^, affecting old animals, resembles atypical forms seen in cattle^30^ and small ruminants^31^. These features confirmed that the sporadic prion disease can occur among cervids. More experimental research is clearly required to confirm whether antler cannibalism has actually led to a strain selection process required for the emergence of a contagious CWD. On 3rd of September 2020, a male reindeer was shot in the Hardangervidda population that later tested positive for CWD. This population also have high levels of antler gnawing (Table 1). Whether this new case is related to the CWD outbreak in Nordfjella or represent an independent emergence is uncertain at this stage.

**Table 2:** A scenario for how contagious CWD may have emerged from a sporadic case of CWD among reindeer in the Nordfjella range, Norway (see Supplementary Note).

Steps	Key	Evidence
1	Sporadic CWD with prions in CNS	Sporadic occurrence of prion disease with prions in CNS well known in humans and livestock. Sporadic CWD with prions in CNS found in moose^28^ and red deer^29^
2	Transfer of prions to antlers	**H1** PrP^Sc^ in disintegrating axons integrated in antler bone matrix. Velvet and growth cartilage innervated from the trigeminal nerve^22^. PrP^Sc^ is found in the trigeminal ganglia and tissues innervated therefrom in sCJD patients^23^ **H2** PrP^Sc^ in antler bone marrow. Antler bone viable and vascularized for months after shedding of the velvet^24^. Bone marrow from sCJD patients high levels of infectivity^25^
3	Strain selection process^34^	Oral exposure to prions can lead to infective gut or mucosa associated lymphoid tissue^35^ **H1**. Mixture of prion conformations enter new (non-CNS) tissue after oral exposure^36,37^ **H2**. de novo emergence when entering peripheral tissues after exodus from the CNS^38^
4	Unique epidemiological conditions	Exceptional epidemiological conditions and repeated exposure giving ‘training of prions’ due to antler cannibalism documented in this study
5	Contagious CWD with prions in CNS and lymphatic tissue	All CWD infected reindeer positive in lymphatic tissue^27^

Steps 1–2 in a hypothetical case of sporadic CWD in a reindeer, while steps 3–5 after oral exposure of other individuals to prions from the hypothetical case. CWD = Chronic Wasting Disease; CNS = central nervous system; sCJD = sporadic Creutzfeldt Jakob disease.

## Conclusions

Our study documented the presence of antler cannibalism in the CWD affected reindeer population of Nordfjella, Norway. The presence of a risk factor in terms of oral transmission from a hypothetical case of sporadic prion disease to other individuals, opens an intriguing possibility to how CWD emerged among the reindeer in Norway (Table 2, Supplementary Note). The affected populations are characterised by poor condition and low recruitment rates. Shed antler consumption of red deer in Spain was affected by how the population management had affected the animals’ condition^32^. Hence, to avoid future emergences of CWD, management actions to prevent continued antler cannibalism should be considered from a precautionary perspective. We encourage further investigation to document the other steps (Table 2) required to transform a sporadic form of CWD to a contagious form of CWD.

## Materials and methods

### Study areas

The main study area was focused on where CWD emerged, which was in the Nordfjella reindeer management area zone 1 encompassing 2000 km^2^. The entire population of 2024 reindeer was culled during the period of August 10, 2017, to May 1, 2018. Nordfjella is mainly a high alpine area located above the treeline. Nordfjella is part of a southern meta-population of wild reindeer in the Langfjella mountains, which is subdivided by human infrastructures, mainly in the form of roads. We also obtained data in the adjacent management area zone 2 (5–600 reindeer; 1000 km^2^) of the Nordfjella, which again is connected to Hardangervidda (8000 reindeer; 8000 km^2^). From the northern metapopulation of wild reindeer, we gathered data from subpopulations in Forollhogna (2000 reindeer; 1700 km^2^) and Rondane (3500 reindeer; 3000 km^2^).

### Data

An overview of data sources is given in Table 1. The main data were derived from the 238 photographed reindeer heads during marksmen culling in February 2018, when the entire reindeer population in Nordfjella zone 1 was eradicated. We also derived historical data from photos of herds in the Nordfjella population in 1984 and 2009 to enable analysis of the temporal development of level of antler gnawing. We similarly derived photos and videos of reindeer herds from Hardangervidda, Forollhogna and Rondane. Main movie 1 was taken from a GPS-marked reindeer with a mounted camera on the collar in Nordfjella zone 2. Main movie 2 and pictures for Fig. 2 were taken using the Reconyx Ultrafire XP9 camera traps (Reconyx, USA) that were placed at the artificial salt licks in Nordfjella zone 2.

### Scoring of antler gnawing

We measured the extent of antler gnawing using a qualitative scale of 0 to 4 when we had antlers from dead animals at hand, as for Nordfjella population in 2018 (Supplementary Figure 2). We used a qualitative scoring method because the photographs were taken from different angles, making it difficult to objectively measure the antlers' length. In addition, the individual variations in antler morphology were large. Most males had already shed antlers at that time of the year (February), where 18.1% of all the individuals had an antler on only one side. For a subset of the individuals (96 females and 39 males) from Nordfjella in 2018, we estimated the age based on tooth eruption and reading the annuli in the tooth cementum, while following standard procedures of the National Monitoring Program for Cervids. When scoring antler gnawing visually based on photos or videos of live animals from a distance (Supplementary Figure 3), we pooled categories 0–1 as it was not possible to determine the difference between no or low level of gnawing.

- 0—None: No sign of gnawing.
- 1—Low level: Only antler tine ends were gnawed.
- 2—Medium level: Antler tines were clearly shortened (markedly gnawed).
- 3—High level: Only the main antler beams were left, where the tines were shorter than 2 cm or absent (extensive gnawing).
- 4—Extreme level: Only a stump was left after gnawing.

### Statistical analysis

For the 2018 data from Nordfjella, we ran basic linear models using the scoring values as the response variable, while sex and age were treated as predictors using R (v3.6.2). Even though our scoring was qualitative, using regression models is still appropriate^33^. The benefit of using an ancova-like model with sex and age as predictors, rather than a simpler correlation analysis, is that both sexes can be included in the same model with more statistical power, and that we can test for the interaction between age and sex. For analyses of variation in level of gnawing from different periods (1984, 2009, 2018) in Nordfjella, we pooled into categories low (score 0–1) and high (2–4) to enable comparison of data from different sources. Due to a binary response, we used logistic regression allowing for comparison of estimates for different time periods, rather than just testing for an overall effect using e.g. a chi-square test.

### Ethics statement

This paper only contain data gathered for other purposes, and from using non-invasive methods.

## Electronic supplementary material

Below is the link to the electronic supplementary material.

Supplementary Information 1.

Supplementary Video 1.

Supplementary Video 2.

## Data Availability

All data will be made available in a public repository.
