# Supplementary material for: Antler cannibalism in reindeer
Source: Sci Rep. 2020 Dec 17;10:22168. doi: 10.1038/s41598-020-79050-2 (PMC7747554; doi:10.1038/s41598-020-79050-2)
Supplement: Supplementary file 1 — Supplementary Information 1. [file 41598_2020_79050_MOESM1_ESM.pdf]

Antler cannibalism in reindeer

Atle Mysterud, Bjørnar Ytrehus, Michael A. Tranulis, Geir Rune Rauset, Christer M. Rolandsen, and Olav Strand

Content

- Supplementary Table 1.
- Supplementary Table 2.
- Supplementary Figure 1.
- Supplementary Figure 2.
- Supplementary Figure 3.
- Supplementary video 1 (own attachment)
- Supplementary video 2 (own attachment)
- Supplementary Note.

Supplementary Table 1. Parameter estimates from the linear model analysing scoring of antler gnawing (0-4) as a function of sex and age in reindeer from the CWD-infected population in Nordfjella, Norway. The T-value is the estimate divided by the standard error, and hence, the greater the T-value the more likely there is a difference.

Parameter	Estimate	SE	T	p
Intercept	3.1974	0.1237	25.856	<0.001
Sex (male vs. female)	-0.4847	0.1476	-3.283	0.001
Age (in years)	-0.0678	0.0309	-2.197	0.030

Supplementary Table 2. Parameter estimates from the logistic regression model analysing antler gnawing (low/high) from different time periods in reindeer from the CWD-infected population in Nordfjella, Norway (n = 348). Baseline year was 1984. The 'low' category included scores 0-1 and the 'high' category scores 2-4. The Z-value is the estimate divided by its standard error and hence, the greater the Z-value the more likely there is a difference.

Parameter	Estimate	SE	Z	p
Intercept	2.485	0.520	4.775	<0.001
Year 2009 vs. 1984	-3.450	0.598	-5.773	<0.001
Year 2018 vs. 1984	-5.844	0.633	-9.237	<0.001

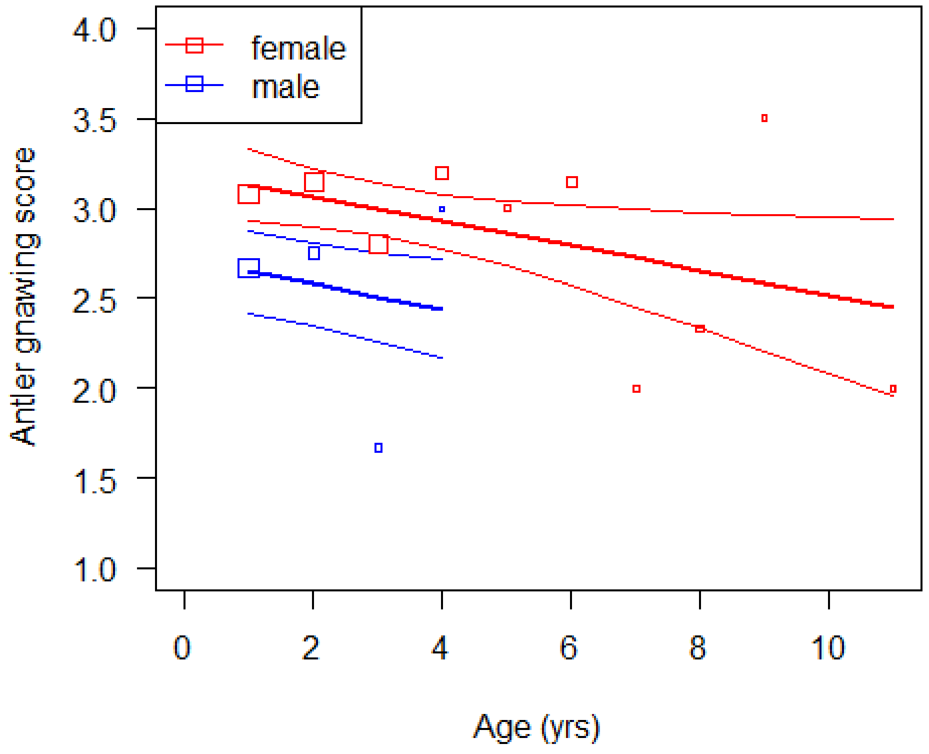

Supplementary Figure 1. The correlation between the severity level of antler gnawing (scale 0 to 4) and age in male (n = 39) and female (n = 96) reindeer in the CWD-infected population in Nordfjella, Norway. The squares are proportional to the (sqrt) sample size. Older males would have shed their antlers during that time of the year in winter (February).

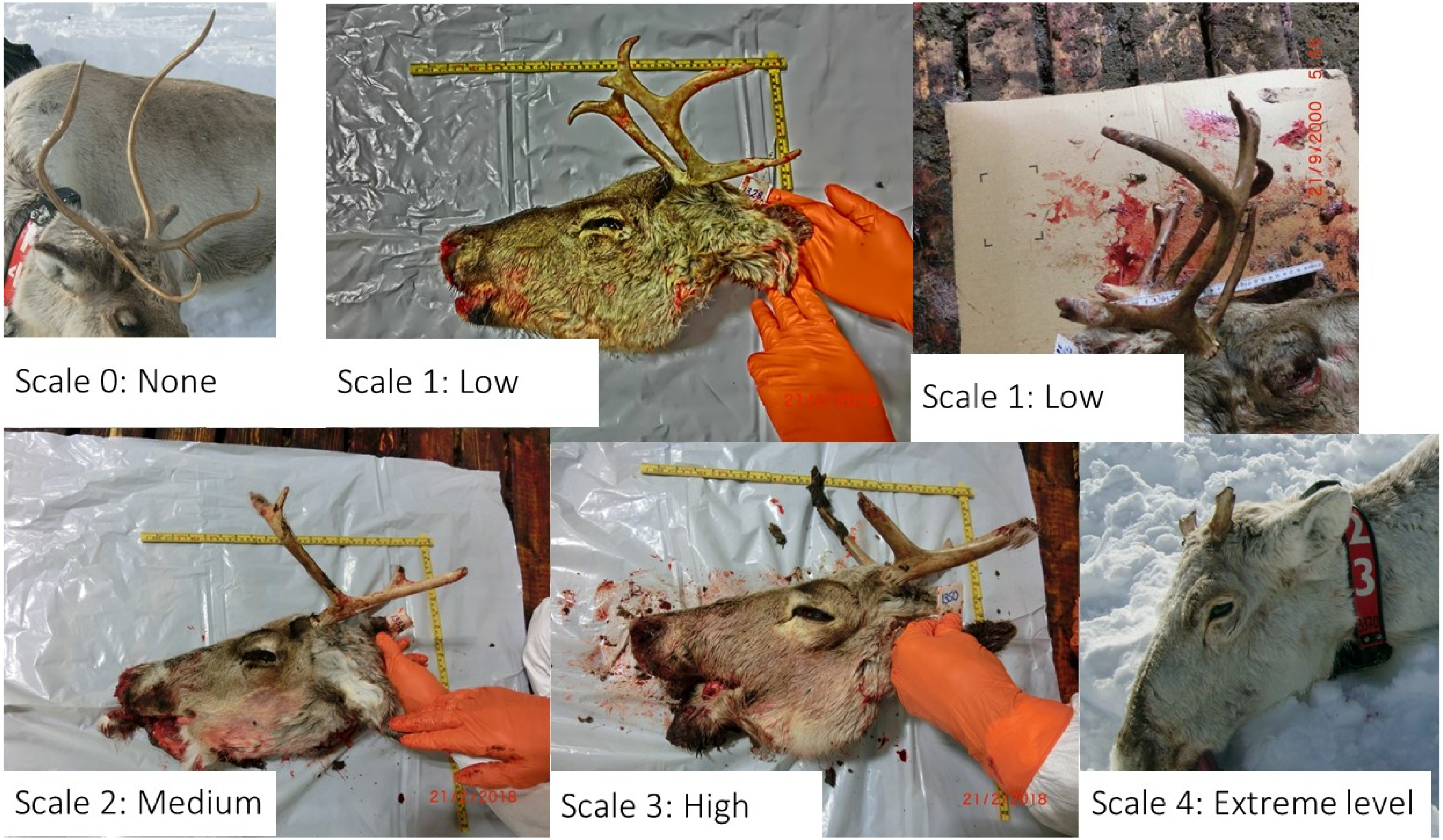

Supplementary Figure 2. An illustration of visual scoring scheme with example photographs of reindeer from Nordfjella, Norway (Photo: Statens Naturoppsyn, SNO).

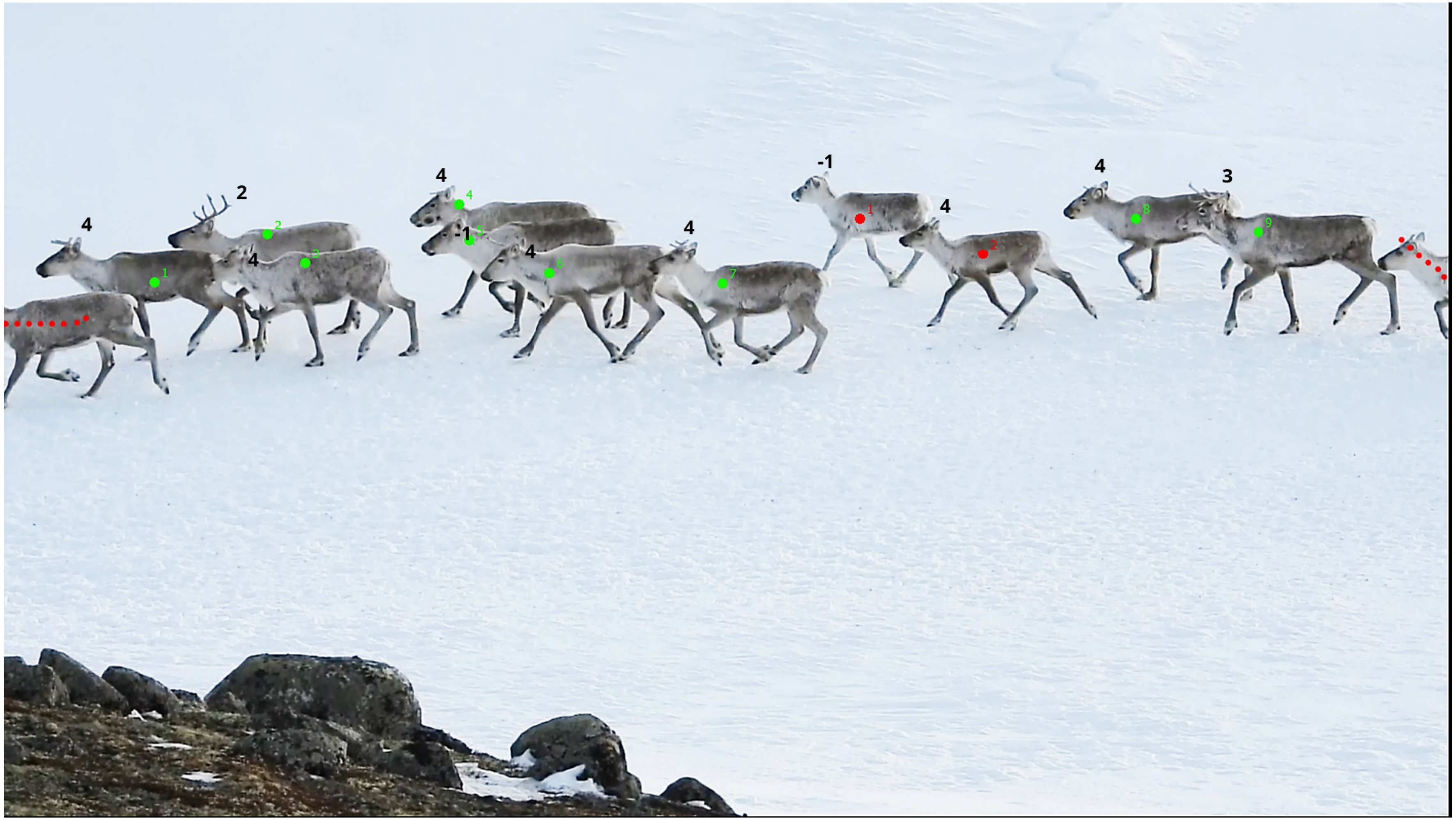

Supplementary Figure 3. Example of how we used pictures to determine level of antler gnawing, based on this example from Hardangervidda, Norway. Animals are scored and numbered as either adult females (green points) or calves (red points) and then given a score (-1 = lacking antler; score 1-4 in black font as described above) (Photo Olaf Brattland, Statens Naturoppsyn, SNO).

Supplementary Note. The hypothesis that contagious CWD may emerge from sporadic CWD.

Prion strain evolution and emergence are only understood at a rudimentary level[1]. CWD prion strain selection are documented to occur in hosts expressing prion protein polymorphisms[2]. Atypical, sporadic forms of CWD has been documented in moose (*Alces alces*) in Norway[3], Sweden and Finland, and a red deer (*Cervus elaphus*) in Norway[4]. Analysis of primary prion isolates has shown that they are heterogeneous. They contain a spectrum of misfolded PrP conformations[3], with one or a few being major conformers, while others are minor components[5,6]. A strain selection process[7] could occur if such a mixture of prion conformations entered a new tissue outside of CNS[5], since conditions for prion propagation offered by the new tissue could allow a minor component prion generated in the CNS to propagate more effectively in the lymphatic system. In other words, the prion would be more lymphotropic and amplified in the peripheral tissues during a late stage prion overflow from the CNS. Another possibility is that new strains might emerge *de novo* when entering peripheral tissues after the exodus from the CNS, excluding the pre-existence of the minor strain components generated in the CNS[8]. Regardless of the model, prion release would most likely be insufficient to readily infect new animals, and thus, be epidemiologically insignificant. However, widespread endocannibalism would result in extraordinary epidemiological conditions, where several flock mates would become directly exposed to the prions via the oral route. This consists of chewing the antlers of a sporadic CWD case with a late-stage prion that would disseminate to peripheral organs. Hence, we propose that a low-level oral exposure due to extensive antler cannibalism, on rare occasions, could lead to the selection of a new lymphotropic prion agent with the capacity to spread under natural conditions.

References

1. Huor, A., Espinosa, J. C., Vidal, E., et al. The emergence of classical BSE from atypical/Nor98 scrapie. *PNAS* **116**, 26853-26862 (2019).
2. Velásquez, C., Kim, C., Haldiman, T., et al. Chronic wasting disease (CWD) prion strains evolve via adaptive diversification of conformers in hosts expressing prion protein polymorphisms. *The Journal of biological chemistry* **295**, 4985-5001 (2020).

3. Pirisinu, L., Tran, L., Chiappini, B., et al. A novel type of Chronic Wasting Disease detected in European moose (*Alces alces*) in Norway. *Emerg Infect Dis* **24**, 2210-2218 (2018).
4. Vikøren, T., Våge, J., Madslien, K. I., et al. First detection of Chronic Wasting Disease in a wild red deer (*Cervus elaphus*) in Europe. *J Wildl Dis* **55**, 970-972 (2019).
5. Angers, R. C., Kang, H. E., Napier, D., et al. Prion strain mutation determined by prion protein conformational compatibility and primary structure. *Science* **328**, 1154(2010).
6. Igel-Egalon, A., Béringue, V., Rezaei, H. & Sibille, P. Prion strains and transmission barrier phenomena. *Pathogens* **7**, (2018).
7. Collinge, J. & Clarke, A. R. A general model of prion strains and their pathogenicity. *Science* **318**, 930(2007).
8. Le Dur, A., Laï, T. L., Stinnakre, M. G., et al. Divergent prion strain evolution driven by PrPC expression level in transgenic mice. *Nature Comm* **8**, 14170(2017).
